# A Novel Optical Method To Reversibly Control Enzymatic Activity Based On Photoacids

**DOI:** 10.1038/s41598-019-50867-w

**Published:** 2019-10-07

**Authors:** Heike Kagel, Frank F. Bier, Marcus Frohme, Jörn F. Glökler

**Affiliations:** 1 Technical University of Applied Sciences, Department of Molecular Biology and Functional Genomics, Hochschulring 1, Wildau, 15745 Germany; 2 Potsdam University, Institute for Biochemistry and Biology, Karl-Liebknecht-Str. 24-25, Potsdam, 14476 Germany

**Keywords:** Inorganic LEDs, Enzymes, Biophysical chemistry

## Abstract

Most biochemical reactions depend on the pH value of the aqueous environment and some are strongly favoured to occur in an acidic environment. A non-invasive control of pH to tightly regulate such reactions with defined start and end points is a highly desirable feature in certain applications, but has proven difficult to achieve so far. We report a novel optical approach to reversibly control a typical biochemical reaction by changing the pH and using acid phosphatase as a model enzyme. The reversible photoacid G-acid functions as a proton donor, changing the pH rapidly and reversibly by using high power UV LEDs as an illumination source in our experimental setup. The reaction can be tightly controlled by simply switching the light on and off and should be applicable to a wide range of other enzymatic reactions, thus enabling miniaturization and parallelization through non-invasive optical means.

## Introduction

Enzymatic reactions can occur spontaneously provided that sufficient amount of substrate is available and no otherwise inhibiting conditions are present. In many settings, such as enzyme kinetic measurements, it is desired to control at least the starting point of the reaction. Generally, this can be achieved by adding the enzyme or substrate, but this is not applicable to assays in which many reactions are to be monitored in parallel or when the handling would require opening and closing of tubes, potentially resulting in a less reproducible assay. In addition, it is also useful to stop the reaction at a defined point in order to correctly measure the end point as well.

**Figure 1:**
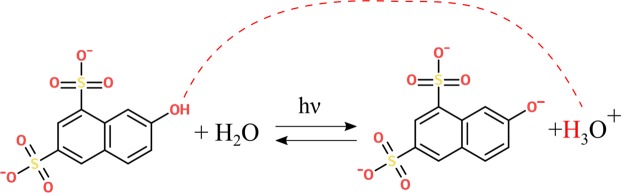
Reversible, light-induced mechanism of G-acid deprotonation.

Thus, it would be highly desirable to initiate and stop such reactions in a non-invasive manner. Typical non-invasive means to control enzymatic reactions are temperature change and irradiation. The former is mostly applicable to reactions that occur at more extreme temperature points involving thermophilic enzymes. The latter requires a receptor or transducer that responds to the radiation in a defined manner. This receptor can either be the enzyme in the biochemical reaction itself^1–3^, the substrate^4,5^ or inhibitor^6–8^. Especially engineering of proteins to become photoresponsive is very difficult to achieve and not applicable to all enzymes, therefor optical control of substrate or inhibitor may represent a more simple alternative. For instance, photocaged reagents can be simply deprotected by a light source. However, this often requires a longer time period in which the deprotection occurs and thus undesirably delays the start of the enzymatical reaction. A more general alternative is the use of a transducer that directly influences the properties of the reaction environment. Many biochemical reactions occur in specific compartments under defined environmental conditions. For example, lysosomes are cellular compartments in which hydrolytic reactions occur that are strongly accelerated under acidic conditions^9^. Therefore, many hydrolytic biochemical reactions should be controllable if the pH can be changed by optical means. A recent publication has explored the concept of “photoswitching” of enzyme activity by a transducer that changes the environment of the reaction^10^. In this case, a photoacid generator (PAG) is used as the radiation-responsive transducer and acid phosphatase (AP) as a model pH-dependent hydrolytic enzyme. As the PAG 2-nitrobenzaldehyde can be rapidly triggered by irradiation, the pH is strongly decreased, thereby activating the acid phosphatase. However, the conversion of the photoacid generator is irreversible and cannot be used to stop the reaction once it has been started. Thus, it would be advantageous to employ a reversible radiation-responsive transducer in order to enable optical control of the enzymatic reaction by pH change over the entire time course in a non-invasive manner. We have identified a reversible photoacid (PA) as a potential radiation-responsive transducer that is able to change the environmental pH. In contrast to a PAG used by Kohse *et al*., a reversible photoacid is not structurally disintegrated upon illumination, but can recombine with a proton after returning to the ground state. Very common families of photoacids comprise phenol, naphthol and pyrene derivatives. The most defining feature for all reversible photo acids is a functional group that donates the proton in an excited state proton transfer (ESPT) process^11^. Depending on various factors the photo-released proton can diffuse and escape the zone of influence of the parent ion, enabling it to react with the surrounding environment. It is also possible that it is re-captured by the parent ion after returning to the ground state S0. The dissociation can be induced by a laser pulse, converting a weak acid into a strong acid^12,13^. This process may occur rapidly within mere pico- to nanoseconds. We employed the photoacid 2-Naphthol-6, 8-disulfonic acid (G-acid) belonging to the family of naphthol derivatives, which is well soluble in water and has a low toxicity. The mechanism of G-acid deprotonation has been well established^11,14,15^ (See Fig. 1).

An optically triggered pH change was demonstrated to occur in a virtually instantaneous manner within nanoseconds^16,17^. It was shown that aqueous solutions could be acidified on a microsecond scale by exciting the photoacids HPTS and G-acid, using a short 347.2 nm laser pulse of 50 ns. Despite having been extensively used to study ESPT, the application of PAs in biochemistry and biology are still very scarce, reviewed in^18^. In order to use a photoacid in a wide range of biochemical applications it must meet certain requirements: It should be soluble in water, have a low toxicity and excitable with commonly available, inexpensive illumination sources, such as LEDs. LEDs have several advantages compared to more conventional illumination sources: They are more efficient, have a long life time, a compact size, and high reliability^19^. Furthermore, they can be easily obtained and the mounting is significantly less complex than that of laser systems. Furthermore, we have chosen an acid phosphatase as a model enzyme. The activity optimum of APs typically lies at an acidic pH of 4.5–5.5^20^. APs non-specifically catalyze the hydrolysis of e.g. monoesters to produce inorganic phosphate under acidic conditions^21^. More specifically, the acid phosphatase from potato (EC 3.1.3.2) used in our experiments is active between pH 4–7^22^, with an activity optimum at a pH of 5–5.3^20,23^. At an alkaline pH of 8 the activity is several orders of magnitude lower%^10^. A standard absorption assay to determine AP’s activity is the p-nitrophenyl phosphate (pNPP) assay^24^. The pNPP assay is a colorimetric assay. The substrate pNPP is hydrolyzed by acid phosphatase into the product p-nitrophenol (pNP) + Pi in purified HPLC grade water:

1 $$pNPP\mathop{\longrightarrow }\limits^{{\rm{Acid}}\,{\rm{Phosphatase}}}pNP+{P}_{i}$$

The chromogenic reaction product, pNP turns yellow (absorption at *λ* max = 405 nm) at a pH of 12 and can be detected using an microtiter plate reader^24^. Due to its pH dependent activity this enzyme is a good choice to demonstrate a controlled, light induced pH switching. Optical control of the pH can be achieved by using reversible photoacids^25–27^ to induce a reversible pH jump. A photoacid is an aromatic alcohol that transforms into a strong acid upon irradiation^28^ and undergoes ESPT in this process^13,29,30^. The acidity enhancement after excitation is typically on a scale of a factor of 10^6^–10^8^ thus decreasing the *pK*_*a*_ by 6–8 units^31^. One important challenge is the solubility of photoacids in aqueous solvents to be useful in typical biochemical reactions. With the work presented here, we demonstrate a reversible control of an enzymatic reaction using a novel system based on a reversible photoacid.

## Results

### Influence of pH and G-acid on Enzyme Kinetics

Acid phosphatase’s activity strongly depends on the pH, with an activity optimum at a pH of 5, whereas at a pH of 8 the activity is several orders of magnitude lower [for detailed pH dependent activity of acid phosphatase see Supplementary Information]. At a pH of 12, G-acid slightly absorbs at 405 nm, causing an offset by 0.14 absorption units, but enzymatic activity is not inhibited by G-acid [Supplementary Information, Fig. S1]. All experiments were conducted using a high (0.21 U/ml) and a low (0.12 U/ml) enzyme concentration. Enzymatic activity was not found to be inhibited by G-acid in the concentration of up to 700 *μ*M. After verification that G-acid has no inhibitory effect on acid phosphatase the citrate buffer was replaced by HPLC water, making G-acid the main buffering component. This maximizes the effect of the protons generated by the photoacid upon excitation.

### Illumination assay

**Figure 2:**
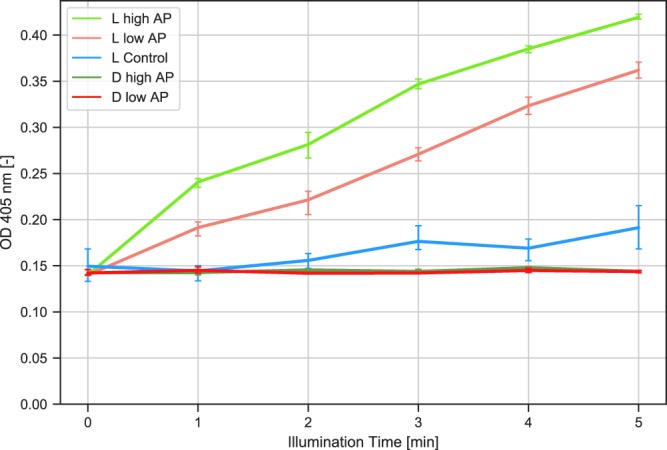
Absorption at 405 nm vs. light exposure times 1–5 min at 365 nm. The total incubation time for all samples is 10 minutes, only illumination time varies from 1–5 min. Enzyme concentrations were 0.12 U/ml (low AP), 0.21 U/ml (high AP) and a control without enzyme containing G-acid and pNPP only (Control). L indicates light; D indicates absence of illumination (darkness).

Before illumination assays were performed, potential enzyme inhibition by illumination with 3 W, 365 nm high power LEDs was tested in a standard assay, by illuminating the sample. Samples were incubated at a pH of 5 in citric acid buffer under conditions identical to the light-controlled experiments. UV light did not affect the enzyme’s activity [Supplementary Information, Fig. S2]. Two different illumination protocols were conducted to demonstrate the reversible control. (1) Continuous illumination assay: Samples were illuminated for 1–5 minutes and each sample was left in the dark afterwards to an overall incubation time of 10 minutes respectively. (2) Cycled illumination assay: Samples were illuminated for 1–5 minutes switching the light on and off alternatingly. Two different cycled assays were performed: One with one minute switching time and another with 30 seconds switching time and hence twice the number of switches in a given interval. All samples were left in the dark after illumination to an overall incubation time of 10 minutes and had the same cumulative 365 nm UV light exposure time. Samples without illumination were incubated for 1–10 minutes in a standard assay [Supplementary Information, Standard assay]. The first experiments were conducted under continuous illumination lasting for 1–5 minutes (Fig. 2). The total incubation time for all samples was 10 minutes, differing in light exposure time only.

**Figure 3:**
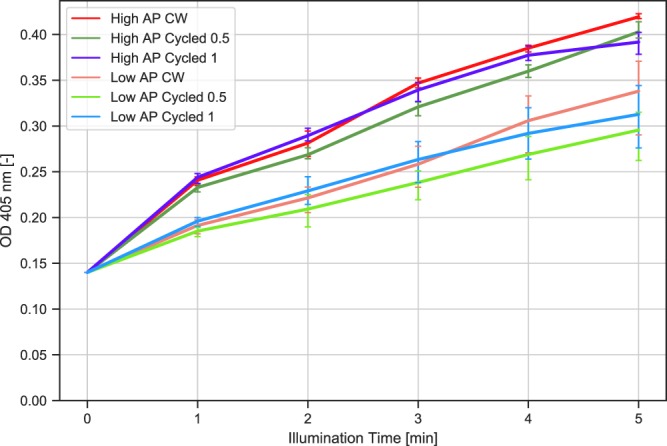
Comparison between continuous light vs. cycled light exposure times. Absorption was measured at 405 nm, exposure was at 365 nm from 1–5 minutes with an total incubation time of 10 min for all samples. Enzyme concentrations were 0.12 U/ml (low AP) or 0.21 U/ml (high AP). CW = continuous illumination; Cycled 0.5 and Cycled 1 indicates intervals of 30 seconds and 1 min.

Acid phosphatase was not found to be active at a pH of 8 in darkness for both enzyme concentrations. A slight rise in absorption due to UV irradiation in absence of enzyme is recorded, indicating a decay of the substrate pNPP due to light exposure. Values obtained from samples comprising G-acid exposed to UV light correlate well with applied enzyme concentrations. In another setup we illuminated the samples in cycles with intervals of one minute and 30 seconds in direct comparison with continuous illumination (Fig. 3).

Exposure times for all three illumination setups resulted in very similar enzyme activities. As the common factor is the total illumination time, it can be safely concluded that the acid phosphatase remains active even under changing pH conditions and that the enzymatic reaction can be reversibly controlled in a rapid and stringent manner.

## Discussion

In our experiments we could demonstrate that the activity of acid phosphatase was light-dependent only in the presence of the optical transducer G-acid. The activity measured by pNPP correlated with the amount of enzyme used with low error margins (Fig. 2). In addition, the amount of product formation clearly corresponded to the duration of the illumination time and not total incubation time that included the dark phase. Most importantly, the product formation remained strongly dependent on the total illumination time even if intermediate switching with intermediate dark phases was performed (Fig. 3). The same also applies to the two different cycling speeds applied in our experiments. Therefore, it is clear that the enzymatic activity measured in our experiments is controlled by illumination and reversibly transduced by G-acid by changing the pH in the reaction. We concede that a major drawback of this experimental setup is that the exact pH cannot be determined online. Yet, many pH-indicators, such as fluorescein or other fluorescent dyes undergo photobleaching^32–34^. We have therefore concluded that an online absorption or fluorescence-based assay is difficult to establish as longer illumination time is required for our experimental setup. Furthermore, we have found that additional dyes will most likely interfere with the spectrum used for excitation and/or substrate turnover detection. Small sample volumes of 100 *μ*l typical for biochemical reactions are not compatible with standard pH electrodes. We have experienced that ISFET-based pH electrodes, although suitable for small volumes and rapid measurements are very light sensitive, thus giving erroneous readings under illumination. Using a micro- pH glass electrode would be generally possible, however those have a relatively long response time^35^. Although not directly comparable, we have therefore resorted to estimate the achieved pH by comparison with enzyme kinetic recorded at different defined pH values for low and high enzyme concentrations [Supplementary Information, Figs S3 and S4 Acid Phosphatase (0.21 U/ml) + 100 *μ*M pNPP + 700 *μ*M G-acid incubated in 45 mM citrate acid buffer at pH values from 4–8]. The reaction pH for the illumination assays is comparable to a reaction conducted at pH 6.5 using citric acid as a buffer. Thus the pH in solution is likely lowered by at least 1.5 pH units. In order to achieve a significant photo-induced pH change by G-acid, residual buffering components had to be reduced to a minimum to avoid proton scavenging. The feasible photo-induced pH jump depends on the specific composition of the system. More specifically, pNPP has a *pK*_*a*_ = 7.1^36^. Hence, to minimize buffering effect of pNPP we only used 100 *μ*M, partially resulting in substrate limitation in the standard assay. Future integration of a dedicated online pH measurement could further be used to finetune the optical pH control by a feed-back mechanism In addition, the enzyme itself may comprise buffering groups and is usually supplied in a stock solution with buffering components for storage. Therefore, it is recommended to obtain enzymes at high stock concentrations in order to avoid carry-over of larger buffer quantities from the stock solution. [Supplementary Information, Figs S5 and S6]. To achieve a maximum excitation of G-acid, components have to be chosen carefully, compromising between detection of enzymatic activity by absorption and managing a sufficient, photo-induced pH jump. As most commercially available LEDs do not readily emit light at the optimum excitation wavelength of G-acid at 347 nm, we resorted to less optimal but comparably inexpensive LEDs emitting at 365 nm. Generally, other components present in the experimental setup must not absorb strongly at the applied excitation wavelength of G-acid. The photo-induced pH jump can be further optimized by using a photoacid with a larger ΔpH, one group of photoacids with a significantly large ΔpH is called “Super photoacids”^13,37,38^ and could be potentially applied. To facilitate the photoacid’s excitation, e.g. by using LEDs, it is preferable if the photoacid’s absorption is in the visible region. By using nano- LEDs or lasers as an excitation source the whole setup can be further optimized and miniaturized. In addition, digital micromirror devices (DMD) can be used to selectively and simultaneously control illumination of individual pixels in vast arrays. Both enzyme and G-acid exhibited a high robustness in our illumination experiments and remained active, irrespective of the number of applied light switching cycles. Early manual implementations of the described assays have resulted in strong variations due to heat emanating from LEDs and less reproducible arrangement of the samples. As most commercially available LEDs do not readily emit light at the optimum excitation wavelength of G-acid at 347 nm, we resorted to less optimal but comparably inexpensive LEDs emitting at 365 nm. Temperature rises by circa 4 °*C* when being illuminated for 5 minutes in this setup, which should be considered as some enzymatic reactions strongly depend on temperature [Supplementary information Fig. S7 for detailed temperature monitoring]. Finally, our self-constructed high power LED standard microtiter 96 well array^39^ was found to be suitable for conducting the photoswitching experiments, by ensuring high reproducibility and low error margins. Other examples for interesting enzymes with a pH dependent activity could be Laccase, Catalase or Polyphenol Oxidase^10^.

## Conclusion

In this seminal study we could demonstrate the feasibility of a light-induced, reversible control of the enzymatic activity of acid phosphatase in a novel non-invasive manner. The main advantage of optical vs. thermal control of biochemical reactions is the rapid manner by which conditions can be changed and the highly precise spatial resolution that can be achieved without complex hardware and comparably low energy consumption. Reversible photoacids thus offer a new way to transduce this localized pH control, making them highly attractive for miniaturizable, non-invasive and time-resolved control of biochemical reactions, especially in highly parallelized and combinatorial settings such as arrays, microfluidics and emulsions. We propose that reversible photoacids thus have a yet unexplored potential to be used in high throughput applications and automation. We also demonstrated the feasibility to control reversible photoacids using commercially available LEDs, making their application in highly integrated devices and instruments more attractive.

## Materials and Methods

All experiments were conducted at 40 °*C* in black 96 well microtiter plate plates with transparent flat bottom (BRAND GMBH + CO KG, Wertheim, Germany) in triplicates. All data generated or analyzed during this study are included in this published article (and its Supplementary Information files).

### Absorption of compounds

Absorption spectra were recorded with the Tecan Infinite® 200 PRO multimode plate reader. Dilutions of G-acid (Carbosynth Ltd, Compton, UK) and all other components were performed in 45 mM TRIS-HCL buffer (Roth GmbH + Co. KG, Karlsruhe, Germany) at a pH of 8. For detailed absorbance spectra at a pH of 8, which was the starting pH for all illumination assays, see Supplementary Information, Fig. S6. General chemicals used in the laboratory were bought from Sigma Aldrich (Taufkirchen, Germany). TCI Chemicals (USA) or Carl Roth GmbH + Co. KG (Karlsruhe, Germany) with the purity grade “for analysis”.

### Enzyme kinetics at different pH values

Acid phosphatase from potato (EC 3.1.3.2) was purchased from Sigma Aldrich (Germany) as lyophilized powder with 3.6 U/mg solid and was stored at −20 °*C*. For each experiment acid phosphatase was prepared freshly in cold HPLC grade water (Carl Roth GmbH + Co. KG, Karlsruhe, Germany), as recommended by supplier. Stock concentration was 30 u/ml, this was further diluted to the enzyme concentration needed for the experiment. All experiments were conducted at 40 °*C*. Acid phosphatases dephosphorylate phosphate groups from phosphate esters under acid conditions. Standard assay was conducted with 100 *μ*M pNPP, and a low and a high acid phosphatase concentration respectively. Low acid phosphatase concentration was 0.12 U/ml and high acid phosphatase concentration was 0.21 U/ml. Experiments were conducted in 45 mM citrate acid buffer with a pH ranging from 4–6.5. All experiments were conducted with 100 *μ*l per well at 40 °*C* and stopped with 100 *μ*l 3 M NaOH after the incubation time, resulting in an overall volume of 200 *μ*l per well. For blank, enzyme was added after 100 *μ*l 3 M NaOH. Samples were incubated for 1–10 mins and stopped each minute. To test potential inhibition of G-acid on the enzyme an assay was conducted with 100 *μ*M pNPP, 700 *μ*M G-acid and low and high AP concentration respectively. Experiments were conducted in 45 mM citrate acid buffer with a pH from 4–6.5. All experiments were conducted with 100 *μ*l per well and stopped with 100 *μ*l 3 M NaOH after the incubation time, resulting in an overall volume of 200 *μ*l per well. For blank, enzyme was added after 100 *μ*l 3 M NaOH. Samples were incubated for 1–10 mins and stopped each minute. Sample absorbance was measured using a plate reader Infinite 200 PRO® (Tecan, Männedorf, Switzerland).

### Illumination assay

For illumination assays, a self-constructed device with 3 W high power 365 nm LEDs, suitable for a 96 well microtiter plate, was used. Standard assay contained 700 *μ*M G-acid, low (0.12 U/ml) or high (0.21 U/ml) enzyme concentration and 100 *μ*M pNPP. Reaction in 45 mM pH 5 citric acid buffer was stopped using 100 *μ*l 3 M NaOH, resulting in a final volume of 200 *μ*l. After the reaction was stopped with 100 *μ*l 3 M NaOH, pH was measured to be 12 for all assays. All samples were incubated at 40 °*C* and experiments were conducted in triplicates. Error bars are included in all diagrams.

## Supplementary information

SUPPLEMENTARY INFO
