# Supplementary material for: A Novel Optical Method To Reversibly Control Enzymatic Activity Based On Photoacids
Source: Sci Rep. 2019 Oct 7;9:14372. doi: 10.1038/s41598-019-50867-w (PMC6779743; doi:10.1038/s41598-019-50867-w)
Supplement: Supplementary file 1 — SUPPLEMENTARY INFO [file 41598_2019_50867_MOESM1_ESM.pdf]

Supplementary Information

A Novel Optical Method To Reversibly Control Enzymatic Activity Based On Photoacids

Heike Kagel, Frank F. Bier, Marcus Frohme, Jörn F. Glökler

Potential Inhibition by G-Acid or Illumination

Potential enzyme activity inhibition by G-Acid or by illumination was tested in the following assays, following a standard assay. First, 700 μM G-acid was added to the standard assay, results are displayed in Figure S1. No inhibition by G-acid can be observed, however a slight increase in absorption by circa 0.14 absorption units is visible

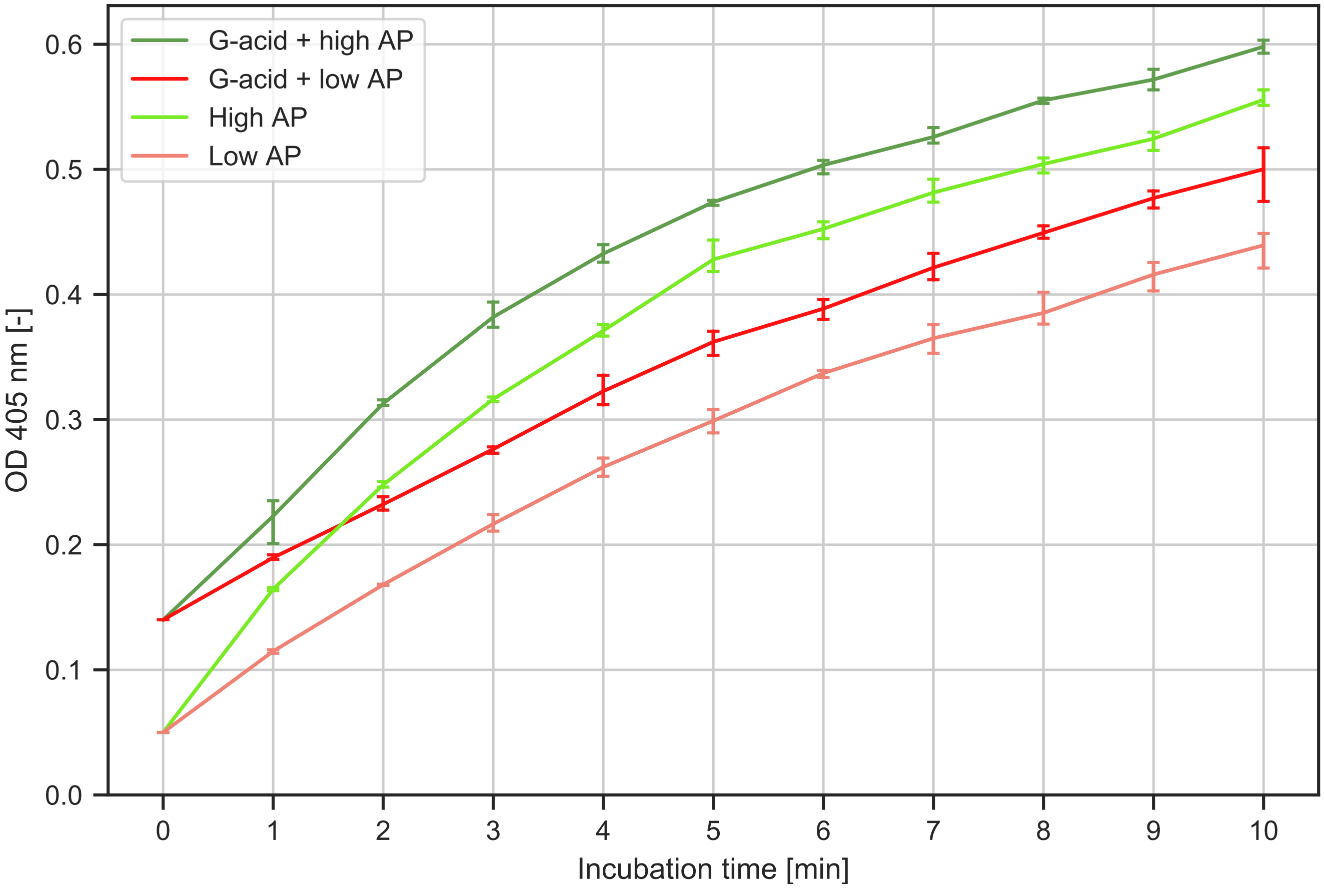

Figure S1. Absorption of acid phosphatase incubated up to 10 minutes in a standard assay with and without G-acid to demonstrate that acid phosphatase is not inhibited by G-acid. Slight increase in absorption at 405 nm can be detected.

Next, potential enzyme inhibition by UV high power LEDs was tested (Figure S2). No enzyme activity inhibition by illumination is observed.

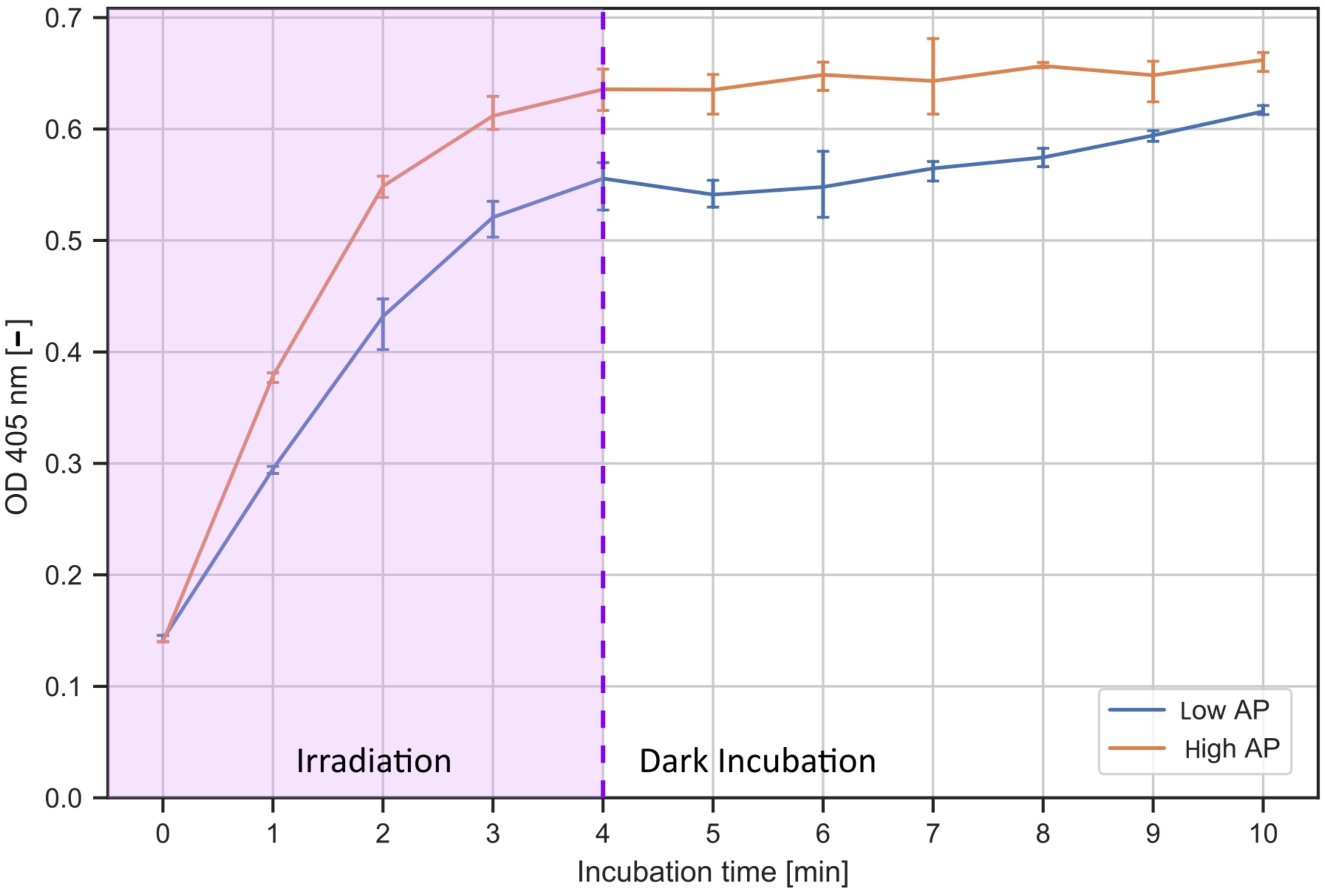

Figure S2. Testing irradiation influence on the enzymatic activity in the absence of G-acid in a standard assay. Samples were illuminated starting at 4 minutes incubation time. The enzymatic activity is not hampered by UV irradiation

Standard Assay Acid Phosphatase

In order to estimate pH of illumination assay an enzyme kinetic with a standard assay was conducted. Reaction was done in 45 mM citrate acid buffer at pH- values ranging from 4.5 – 6.5 and 45 mM TRIS-HCl vuffer for pH 8. Samples were incubated from 1- 10 min at 40°C. For both enzyme concentrations a 100 % enzyme activity is recorded at a pH of 5.5. Standard assay was conducted with 100 μM pNPP, 700 μM G-acid and two acid phosphatase concentrations. Low acid phosphatase concentration was 0.12 U/ml and high acid phosphatase concentration was 0.21 U/ml. Experiments were conducted in 45 mM citrate acid buffer with a pH of 5. All experiments were conducted with 100 μl per well and stopped with 100 μl 3 M NaOH after the incubation time resulting in an overall volume of 200 μl per well. For zero minutes' incubation time, enzyme was added after adding 100 μl 3 M NaOH. Samples were incubated for 1-10 mins and stopped each minute. See Figure S3 for enzyme kinetics for low AP concentration:

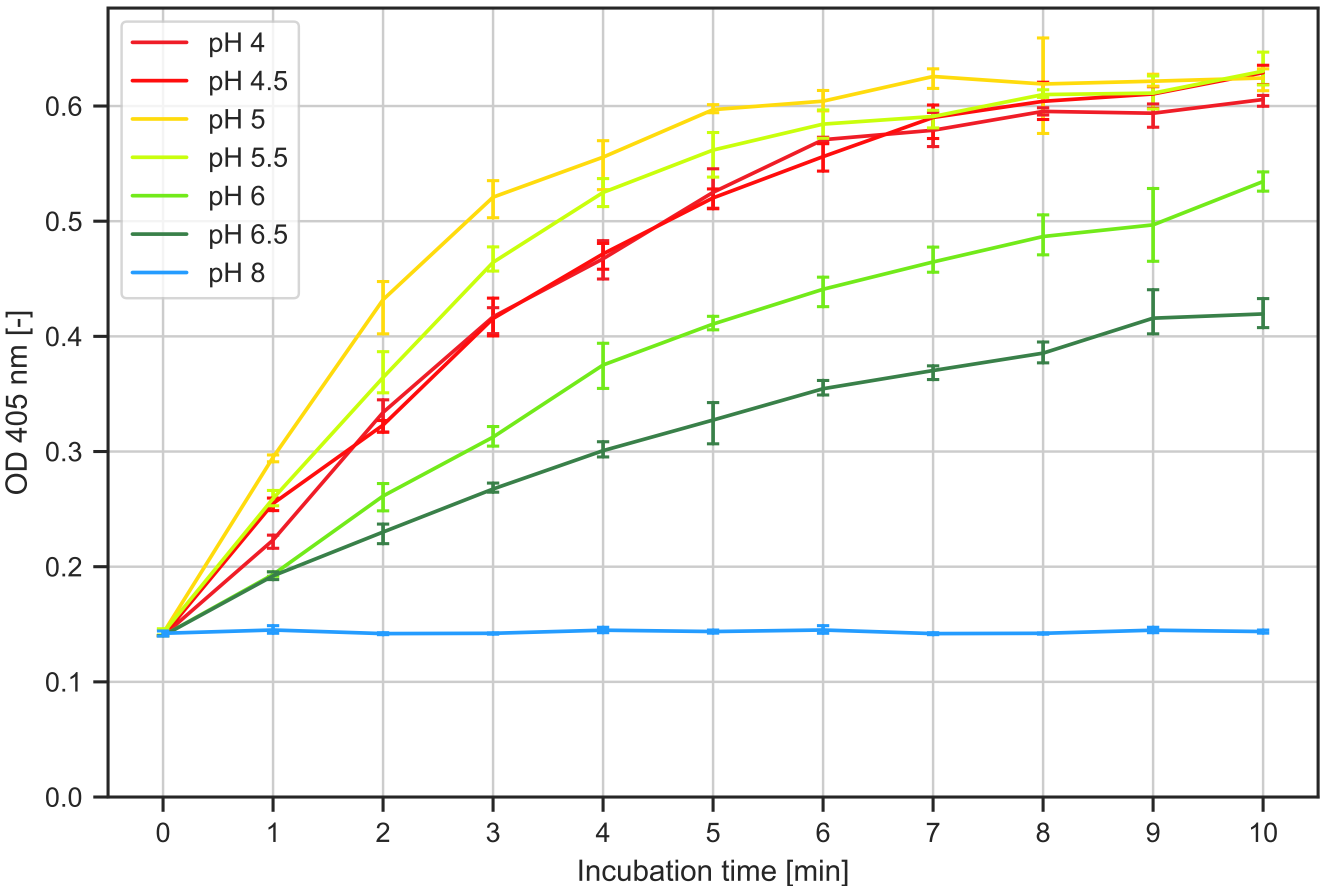

Figure S3. Acid Phosphatase (0.12 U/ml) + 100 μM pNPP + 700 μM G-acid incubated in 45 mM citrate acid buffer at pH values from 4 – 6.5 and 45 mM TRIS- HCl buffer for pH 8

Enzyme kinetics for high AP concentration:

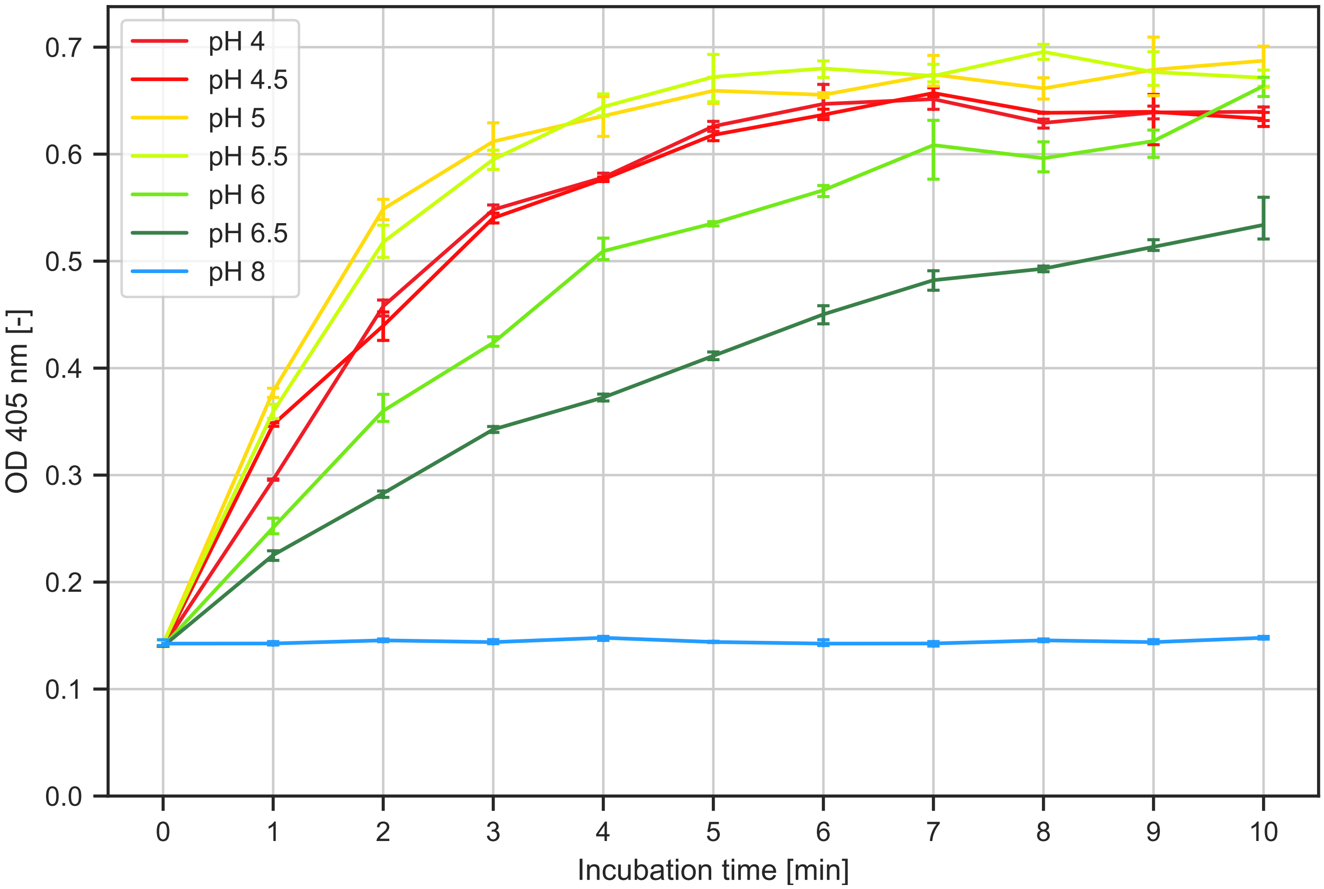

Figure S4. Acid Phosphatase (0.21 U/ml) + 100 μM pNPP + 700 μM G-acid incubated in 45 mM citrate acid buffer at pH values from 4 -6.5 and 45 mM TRIS- HCl buffer for pH 8

PH Dependent Absorption of G-acid, Components Used and Analysis of Temperature during Illumination

The reversible photoacid g-acid absorbs, depending on the pH. G-acid was dissolved in citrate acid buffer (pH 4-6) and TRIS-HCl buffer (pH 7-9) with a concentration of 100 μM. For pH of 12 g-acid was dissolved in HPLC grade water and 3 M NaOH was added

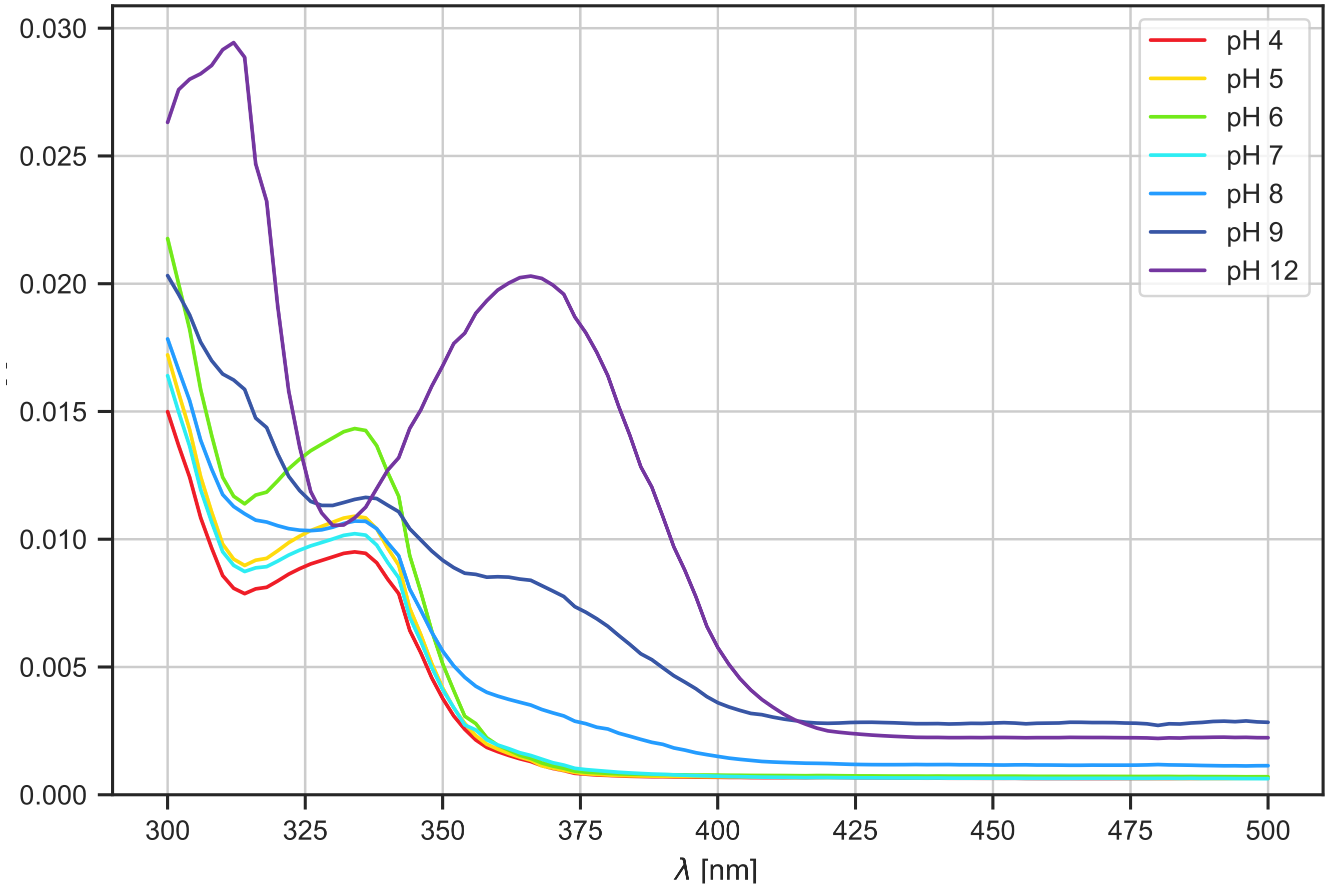

Figure S5. pH dependent absorption of 100 μM G-acid in different 45 mM citrate buffers from pH 4-6, in 45 mM TRIS-HCl buffer (pH 7-9) and at pH of 12 after adding a few drops of NaOH. Spectra of different pH were recorded in a 96 well microtiter plate using the microtiter plate reader. A strong pH dependent absorption of g-acid is detected. The highest absorption is detected at a pH of 12.

The absorption of all components should be taken in to account, Figure S6 displays the absorption at a pH of 8.3 of all components used:

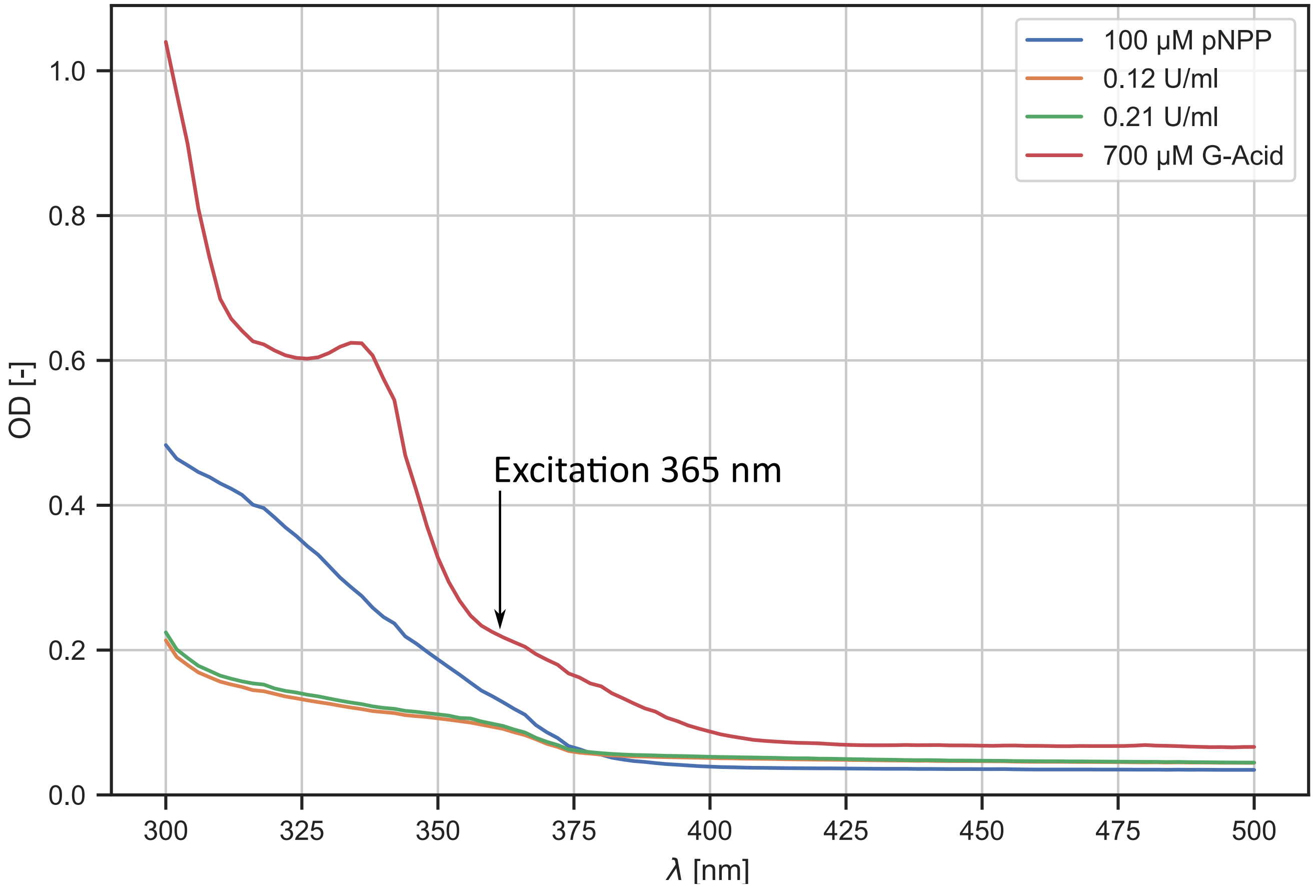

Figure S6. Absorption spectra of all components used at pH 8 (45 mM TRIS- HCl buffer). Red line: 0.12 U/ml acid phosphatase, green line: 0.21 U/ml acid phosphatase, blue line: 100 μM pNPP, purple line: 700 μM G-acid

Temperature change upon illumination was recorded using a 302 K/J Omega from Engineering GmbH Deckenpfronn, Germany in constant illumination mode.

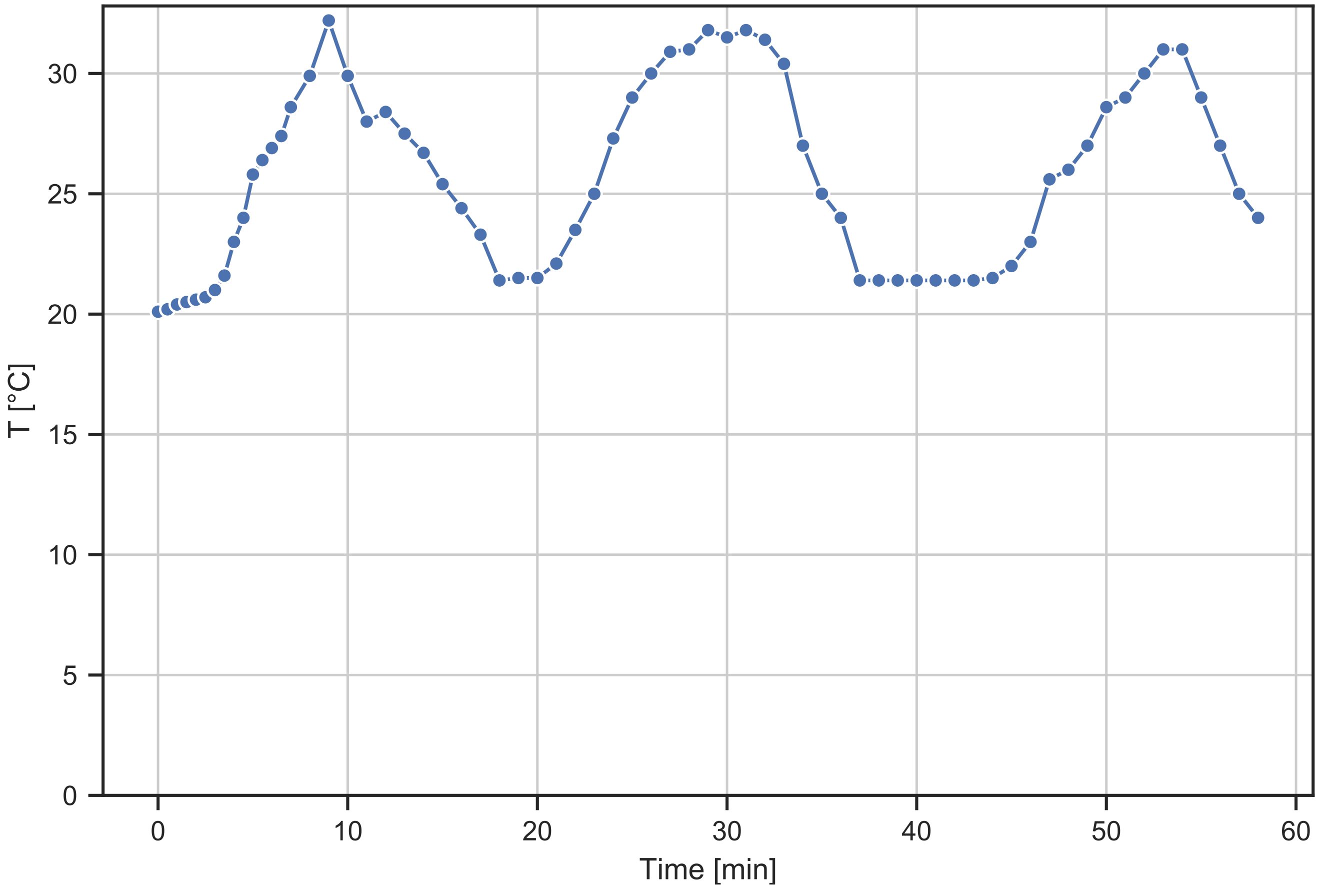

Figure S7. Temperature rise and fall for 7 minute on/off cycles. The temperature rises by 12°C when 100 μl are constantly illuminated for 7 minutes. The temperature rise can be lowered by using a pulsed instead of a constant illumination
